# Supplementary material for: Publication bias in the social sciences since 1959: Application of a regression discontinuity framework
Source: PLoS One. 2025 Feb 14;20(2):e0305666. doi: 10.1371/journal.pone.0305666 (PMC11828420; doi:10.1371/journal.pone.0305666)
Supplement: S1 File — (PDF) [file pone.0305666.s001.pdf]

Supporting information for the manuscript:

Publication Bias in the social sciences since 1959: Application of a regression discontinuity framework [1]

Julia Jerke, Antonia Velicu, Fabian Winter, Heiko Rauhut

Contents

1 Supplementary tables **2**

2 Supplementary figures **4**

3 Supplementary analyses **21**

3.1 Competing for publications in the *QJE* 21

3.2 Analyses with the caliper test 23

[1]Code for the appendix is available upon request.

1 Supplementary tables

List of Tables

S1 Logistic regression of the significance levels 2
S2 Discontinuity estimates, weighted data 3
S3 Overview: Caliper Intervals 24
S4 Cross-sectional caliper counts 25

Table S1. Logistic regression of the significance levels.

	$\alpha = 10\%$		$\alpha = 5\%$		$\alpha = 1\%$	
	(1)	(2)	(3)	(4)	(5)	(6)
year	-0.0002	0.0013	0.0002	0.0017	0.0003	0.0017*
	(0.0010)	(0.0009)	(0.0010)	(0.0009)	(0.0011)	(0.0010)
coeff per article		-0.0020***		-0.0021***		-0.0019***
		(0.0003)		(0.0003)		(0.0004)
Pseudo R^2	0.0000	0.0199	0.0000	0.0181	0.0000	0.0147
N(coefficients)	14091	14091	14091	14091	14091	14091
N(articles)	609	609	609	609	609	609

Note: The dependent variable is a dummy variable indicating whether the coefficient is significant on the 10 percent significance level (column (1) and (2)), the 5 percent significance level (column (3) and (4)) or the 1 percent significance level (column (5) and (6)). The reported coefficients were estimated in a logistic regression and are average marginal effects. Robust standard errors clustered by article are reported in parentheses. * $p < 0.10$, ** $p < 0.05$, *** $p < 0.01$.

Table S2. Discontinuity estimates for the common significance levels, full sample and sub-samples, weighted data.

	$c = 1.64$				$c = 1.96$				$c = 2.58$				N
	(1)	(2)	(3)	(4)	(5)	(6)	(7)	(8)	(9)	(10)	(11)	(12)	coeff.
bandwidth h	[default]	0.8	1.2	1.6	[default]	0.8	1.2	1.6	[default]	0.8	1.2	1.6	articles
1959 - 2018	0.313***	0.259***	0.443***	0.581***	0.274***	0.221***	0.305***	0.385***	-0.025	0.047	-0.162***	-0.249***	12340
	(0.064)	(0.069)	(0.055)	(0.047)	(0.047)	(0.054)	(0.045)	(0.040)	(0.053)	(0.057)	(0.046)	(0.040)	571
	[0.90]				[1.07]				[0.94]				
q1: 1959 - 1973	0.885**	0.253	0.891**	1.124***	0.347	0.087	0.240	0.444*	0.824**	1.442***	0.468	0.108	270
	(0.413)	(0.503)	(0.412)	(0.364)	(0.262)	(0.319)	(0.274)	(0.249)	(0.351)	(0.481)	(0.300)	(0.247)	28
	[1.19]				[1.39]				[1.01]				
q2: 1974 - 1988	0.391*	0.092	0.389*	0.519***	0.253	0.275	0.261	0.254	-0.450*	-0.176	-0.486**	-0.487**	683
	(0.215)	(0.272)	(0.216)	(0.187)	(0.165)	(0.220)	(0.184)	(0.165)	(0.264)	(0.305)	(0.238)	(0.193)	51
	[1.21]				[1.61]				[1.04]				
q3: 1989 - 2003	0.268**	0.231*	0.324***	0.494***	0.416***	0.275***	0.350***	0.430***	-0.200**	-0.045	-0.209***	-0.258***	3876
	(0.112)	(0.125)	(0.101)	(0.086)	(0.075)	(0.102)	(0.084)	(0.074)	(0.082)	(0.096)	(0.081)	(0.070)	198
	[0.98]				[1.51]				[1.17]				
q4: 2004 - 2018	0.323***	0.266***	0.463***	0.586***	0.285***	0.215***	0.299***	0.381***	-0.107*	0.023	-0.156***	-0.252***	7511
	(0.082)	(0.088)	(0.070)	(0.060)	(0.058)	(0.068)	(0.057)	(0.050)	(0.063)	(0.075)	(0.059)	(0.051)	294
	[0.90]				[1.14]				[1.09]				

Note: Each cell reports the results of a single McCrary discontinuity estimation. We weighted the data by the number of coefficients that we extracted per article. In column (1), (5) and (9) we report in brackets the bandwidth that the local linear smoothing procedure determined by default. Additionally, we vary the bandwidth h in the other columns to examine the robustness of our results. We perform the analyses for the 10 percent significance level, indicated by $c = 1.64$, in columns (1)-(3), for the 5 percent significance level, indicated by $c = 1.96$, in columns (4)-(6) and for the 1 percent significance level, indicated by $c = 2.58$, in columns (7)-(9) Standard errors are reported in parentheses. * $p < 0.10$, ** $p < 0.05$, *** $p < 0.01$.

2 Supplementary figures

List of Figures

S1 **Proportion of significance levels over time, 1959 − 2018 (unweighted data)** 5
S2 **Proportion of significance levels over time, 1959 − 2018 (weighted data)** . 5
S3 **Discontinuity plots, $c = 1.64$, varying bandwidth, 1959-2018** 6
S4 **Discontinuity plots, $c = 1.96$, varying bandwidth, 1959-2018** 7
S5 **Discontinuity plots, $c = 2.58$, varying bandwidth, 1959-2018** 8
S6 **Discontinuity plots, $c = 1.64$, varying bandwidth, 1959-1973** 9
S7 **Discontinuity plots, $c = 1.96$, varying bandwidth, 1959-1973** 10
S8 **Discontinuity plots, $c = 2.58$, varying bandwidth, 1959-1973** 11
S9 **Discontinuity plots, $c = 1.64$, varying bandwidth, 1974-1988** 12
S10 **Discontinuity plots, $c = 1.96$, varying bandwidth, 1974-1988** 13
S11 **Discontinuity plots, $c = 2.58$, varying bandwidth, 1974-1988.** 14
S12 **Discontinuity plots, $c = 1.64$, varying bandwidth, 1989-2003** 15
S13 **Discontinuity plots, $c = 1.96$, varying bandwidth, 1989-2003** 16
S14 **Discontinuity plots, $c = 2.58$, varying bandwidth, 1989-2003** 17
S15 **Discontinuity plots, $c = 1.64$, varying bandwidth, 2004-2018** 18
S16 **Discontinuity plots, $c = 1.96$, varying bandwidth, 2004-2018** 19
S17 **Discontinuity plots, $c = 2.58$, varying bandwidth, 2004-2018** 20
S18 **Moving discontinuity estimates for $k = 10$ *years* time windows** 27
S19 **Moving discontinuity estimates for $k = 20$ *years* time windows** 28
S20 **Submissions and acceptance rate over time, 1959 − 2018** 29
S21 **Publication volume over time, 1959 − 2018** 29
S22 **Moving caliper ratios for $k = 15$ *years* time windows** 30

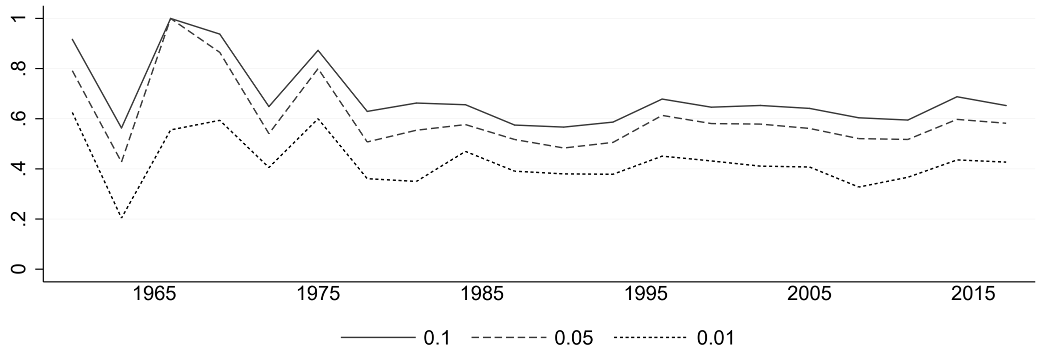

Fig S1. Proportion of significance levels over time, 1959 – 2018 (unweighted data)
We plot the distribution of significance levels over time for the full sample of coefficients, N=14,090. Data is unweighted. The lines represent the share of coefficients reaching a significance level of 10% (solid), 5% (dashed) or 1% (dotted), respectively.

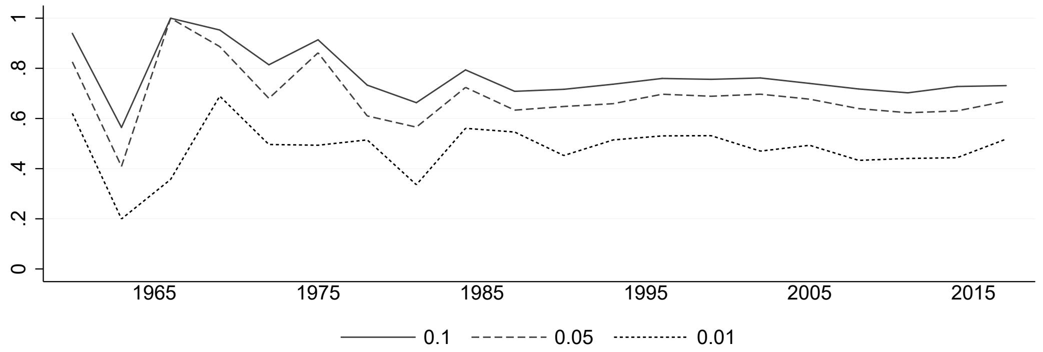

Fig S2. Proportion of significance levels over time, 1959 – 2018 (weighted data)
We plot the distribution of significance levels over time for the full sample of coefficients, N=14.090. Data is weighted. The lines represent the share of coefficients reaching a significance level of 10% (solid), 5% (dashed) or 1% (dotted), respectively.

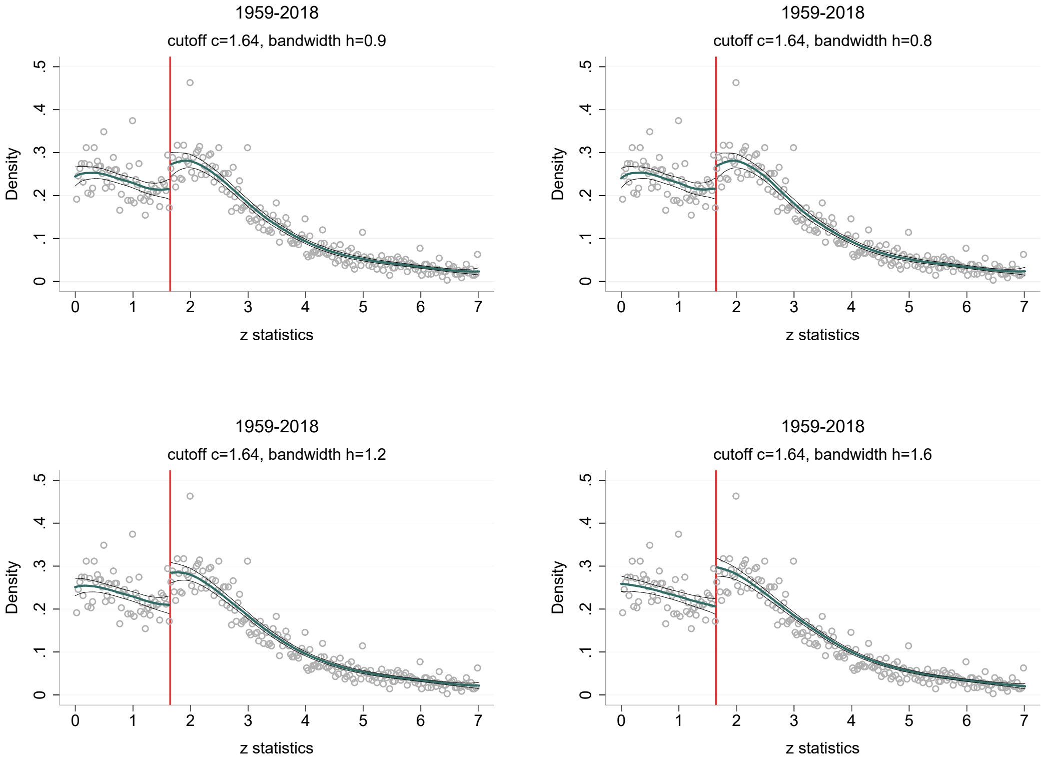

Fig S3. Discontinuity plots, $c = 1.64$, varying bandwidth, 1959-2018

The graphs show the results of the discontinuity estimation by applying the McCrary algorithm for the 10 percent significance level for unweighted data. We vary the bandwidth h of the local linear regression. The upper left graph shows the results for the default bandwidth, the other graphs show the results for $h = \{0.8, 1.2, 1.6\}$. The graphs plot the distribution (grey circles) and the local linear density estimation (emerald line) with the respective 95% confidence band.

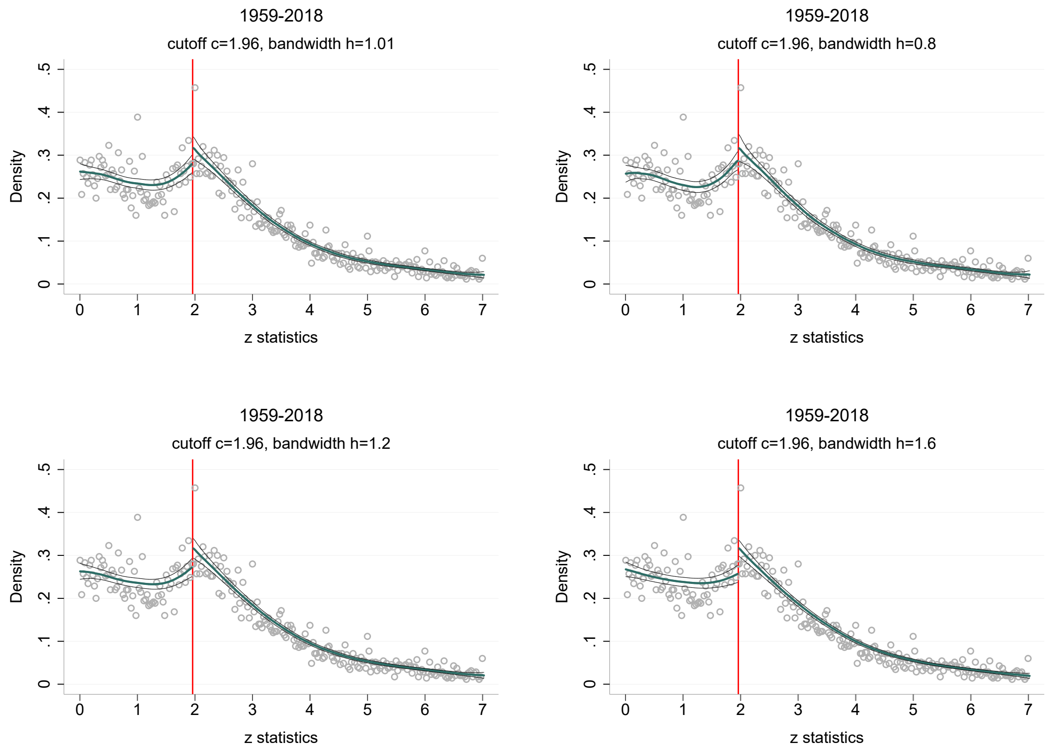

Fig S4. Discontinuity plots, $c = 1.96$, varying bandwidth, 1959-2018
The graphs show the results of the discontinuity estimation by applying the McCrary algorithm for the 5 percent significance level for unweighted data. We vary the bandwidth h of the local linear regression. The upper left graph shows the results for the default bandwidth, the other graphs show the results for $h = \{0.8, 1.2, 1.6\}$. The graphs plot the distribution (grey circles) and the local linear density estimation (emerald line) with the respective 95% confidence band.

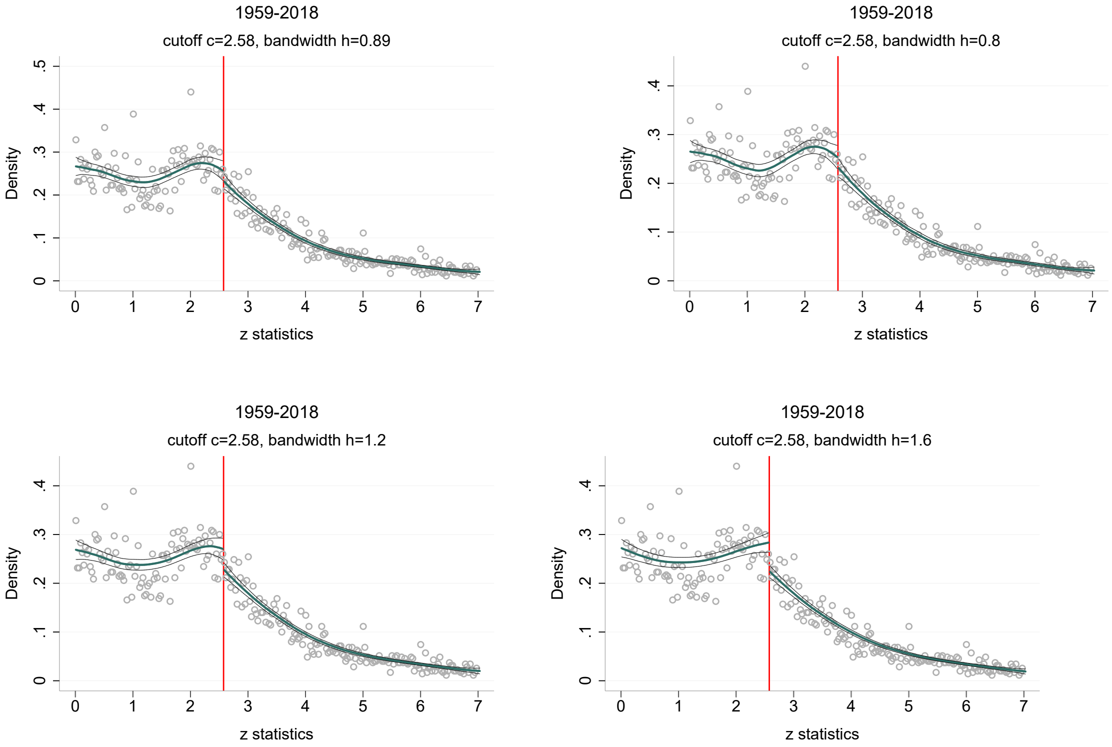

Fig S5. Discontinuity plots, $c = 2.58$, varying bandwidth, 1959-2018
The graphs show the results of the discontinuity estimation by applying the McCrary algorithm for the 1 percent significance level for unweighted data. We vary the bandwidth h of the local linear regression. The upper left graph shows the results for the default bandwidth, the other graphs show the results for $h = \{0.8, 1.2, 1.6\}$. The graphs plot the distribution (grey circles) and the local linear density estimation (emerald line) with the respective 95% confidence band.

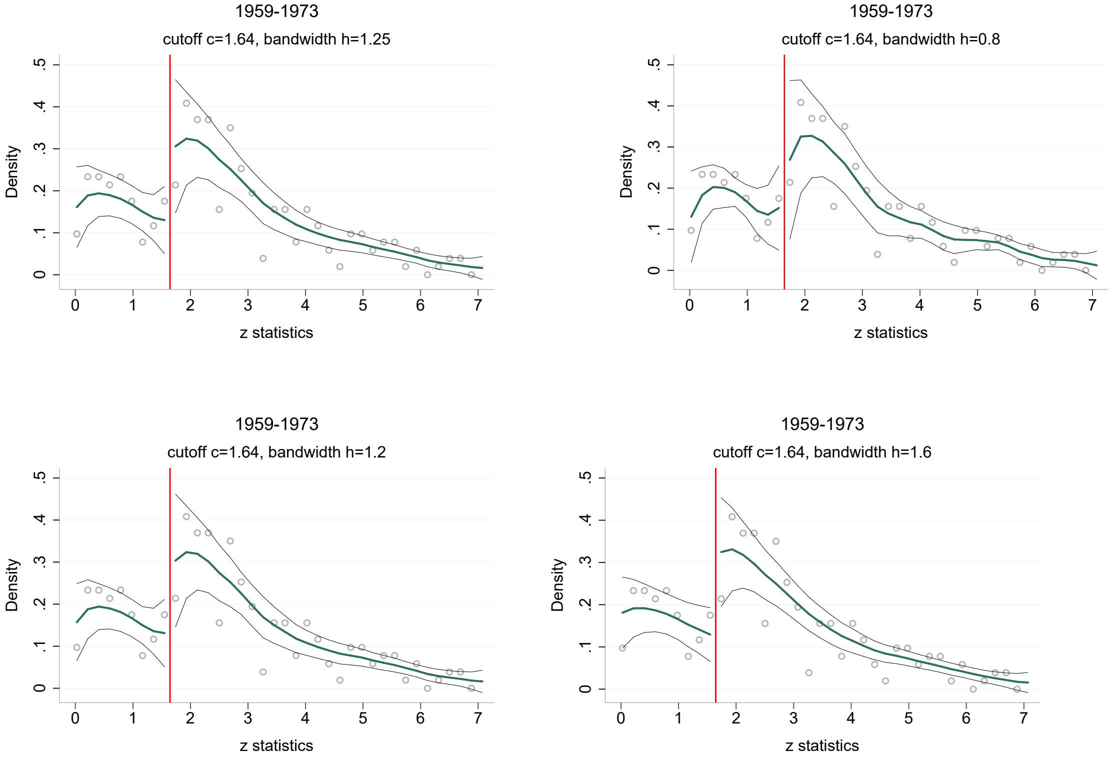

Fig S6. Discontinuity plots, $c = 1.64$, varying bandwidth, 1959-1973
The graphs show the results of the discontinuity estimation by applying the McCrary algorithm for the 10 significance level for unweighted data. We vary the bandwidth h of the local linear regression. The upper left graph shows the results for the default bandwidth, the other graphs show the results for $h = \{0.8, 1.2, 1.6\}$. The graphs plot the distribution (grey circles) and the local linear density estimation (emerald line) with the respective 95% confidence band.

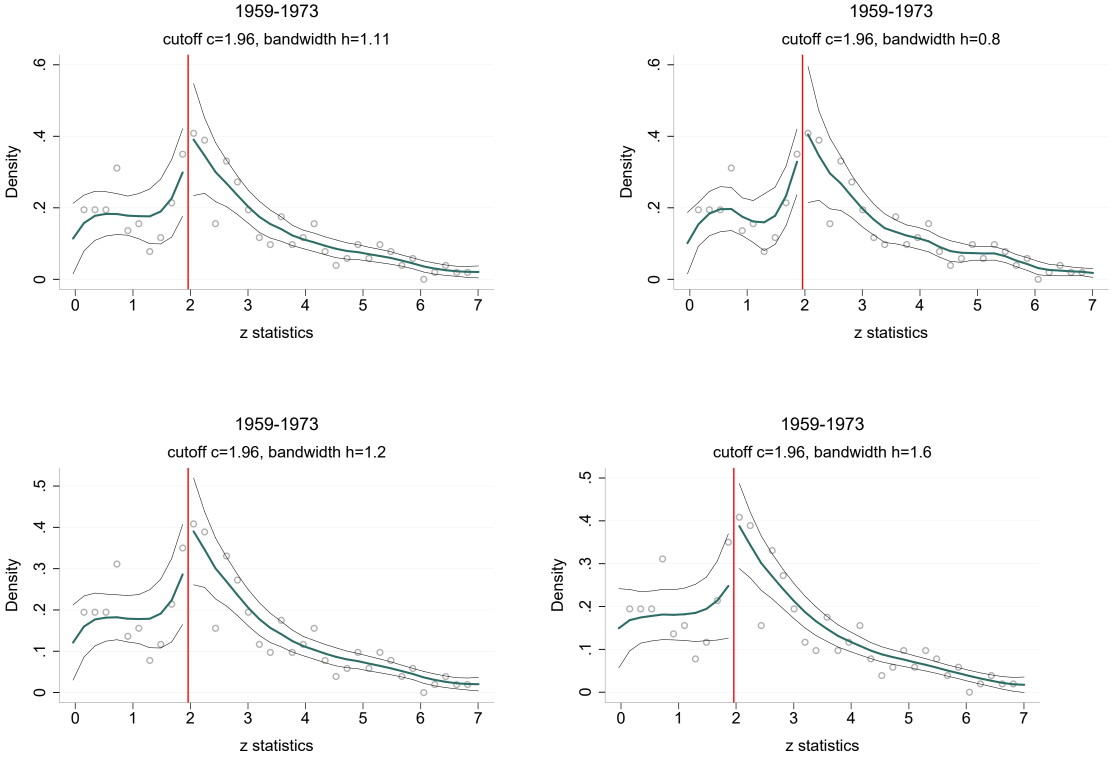

Fig S7. Discontinuity plots, $c = 1.96$, varying bandwidth, 1959-1973
The graphs show the results of the discontinuity estimation by applying the McCrary algorithm for the 5 significance level for unweighted data. We vary the bandwidth h of the local linear regression. The upper left graph shows the results for the default bandwidth, the other graphs show the results for $h = \{0.8, 1.2, 1.6\}$. The graphs plot the distribution (grey circles) and the local linear density estimation (emerald line) with the respective 95% confidence band.

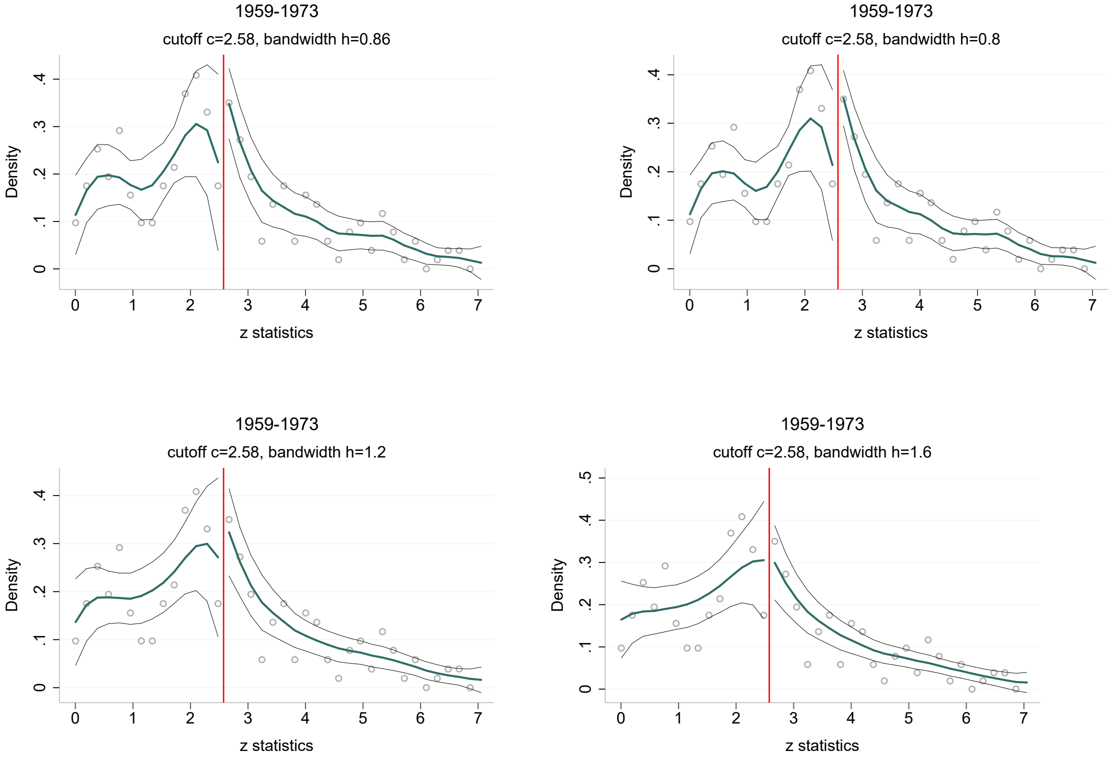

Fig S8. Discontinuity plots, $c = 2.58$, varying bandwidth, 1959-1973
The graphs show the results of the discontinuity estimation by applying the McCrary algorithm for the 1 significance level for unweighted data. We vary the bandwidth h of the local linear regression. The upper left graph shows the results for the default bandwidth, the other graphs show the results for $h = \{0.8, 1.2, 1.6\}$. The graphs plot the distribution (grey circles) and the local linear density estimation (emerald line) with the respective 95% confidence band.

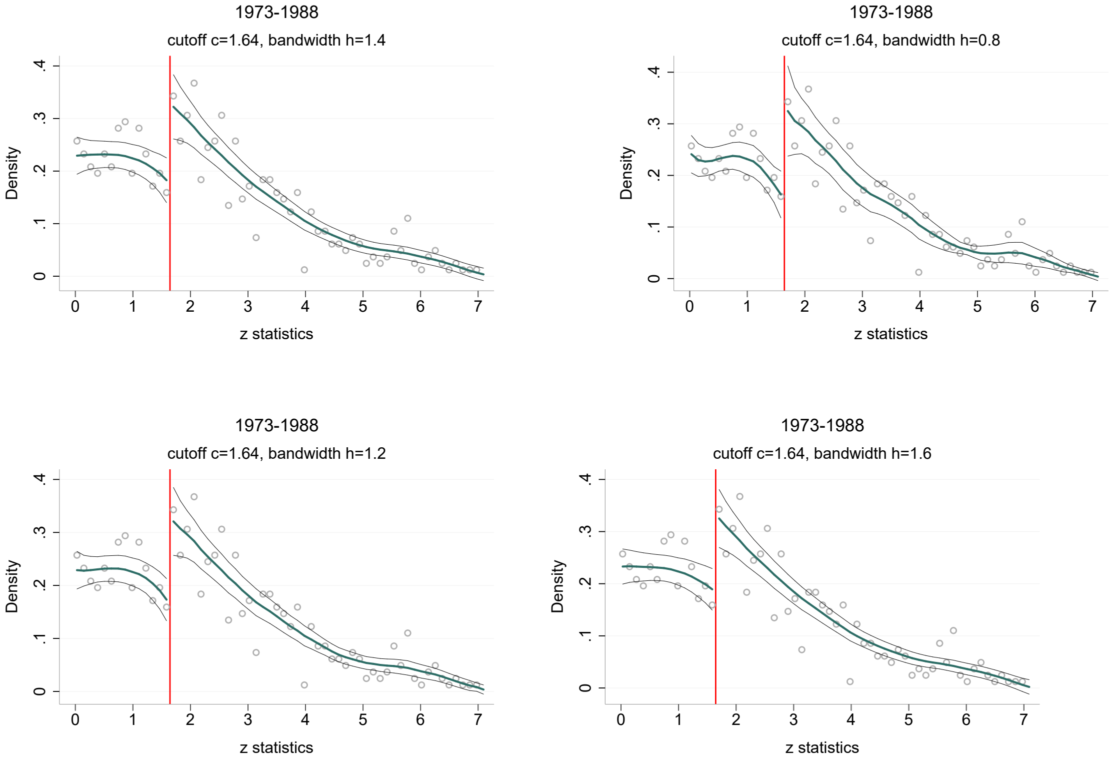

Fig S9. Discontinuity plots, $c = 1.64$, varying bandwidth, 1974-1988
The graphs show the results of the discontinuity estimation by applying the McCrary algorithm for the 10 significance level for unweighted data. We vary the bandwidth h of the local linear regression. The upper left graph shows the results for the default bandwidth, the other graphs show the results for $h = \{0.8, 1.2, 1.6\}$. The graphs plot the distribution (grey circles) and the local linear density estimation (emerald line) with the respective 95% confidence band.

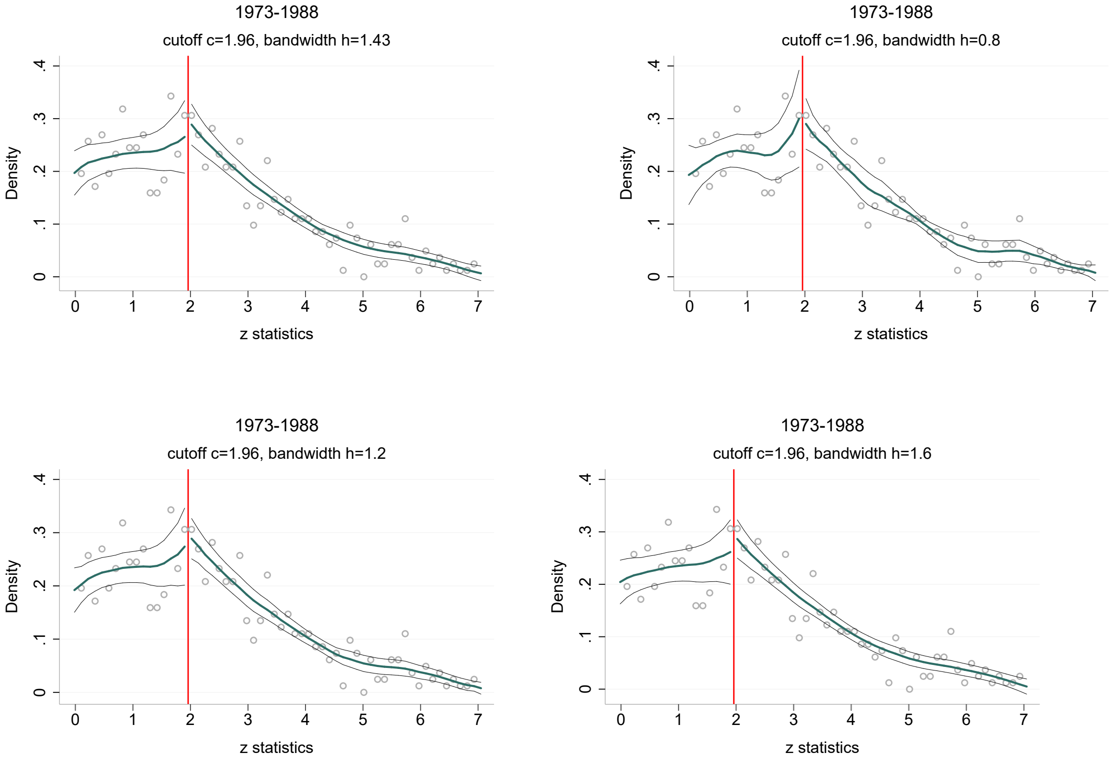

Fig S10. Discontinuity plots, $c = 1.96$, varying bandwidth, 1974-1988
The graphs show the results of the discontinuity estimation by applying the McCrary algorithm for the 5 significance level for unweighted data. We vary the bandwidth h of the local linear regression. The upper left graph shows the results for the default bandwidth, the other graphs show the results for $h = \{0.8, 1.2, 1.6\}$. The graphs plot the distribution (grey circles) and the local linear density estimation (emerald line) with the respective 95% confidence band.

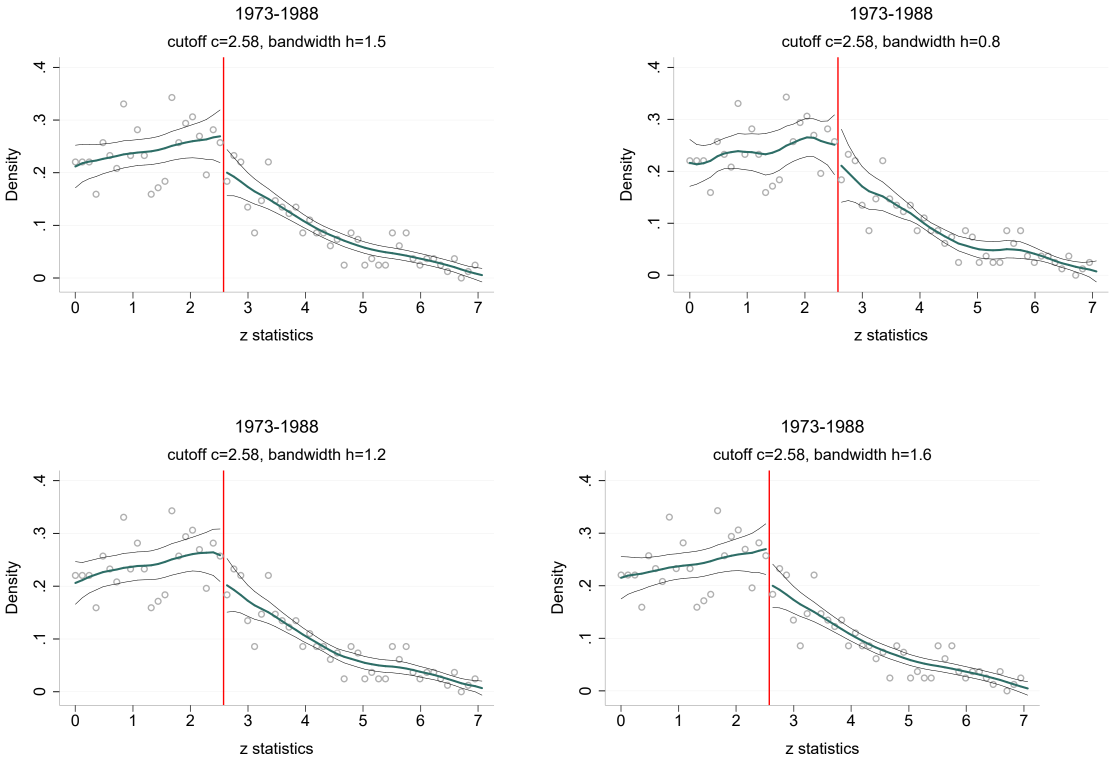

Fig S11. Discontinuity plots, $c = 2.58$, varying bandwidth, 1974-1988.
The graphs show the results of the discontinuity estimation by applying the McCrary algorithm for the 1 significance level for unweighted data. We vary the bandwidth h of the local linear regression. The upper left graph shows the results for the default bandwidth, the other graphs show the results for $h = \{0.8, 1.2, 1.6\}$. The graphs plot the distribution (grey circles) and the local linear density estimation (emerald line) with the respective 95% confidence band.

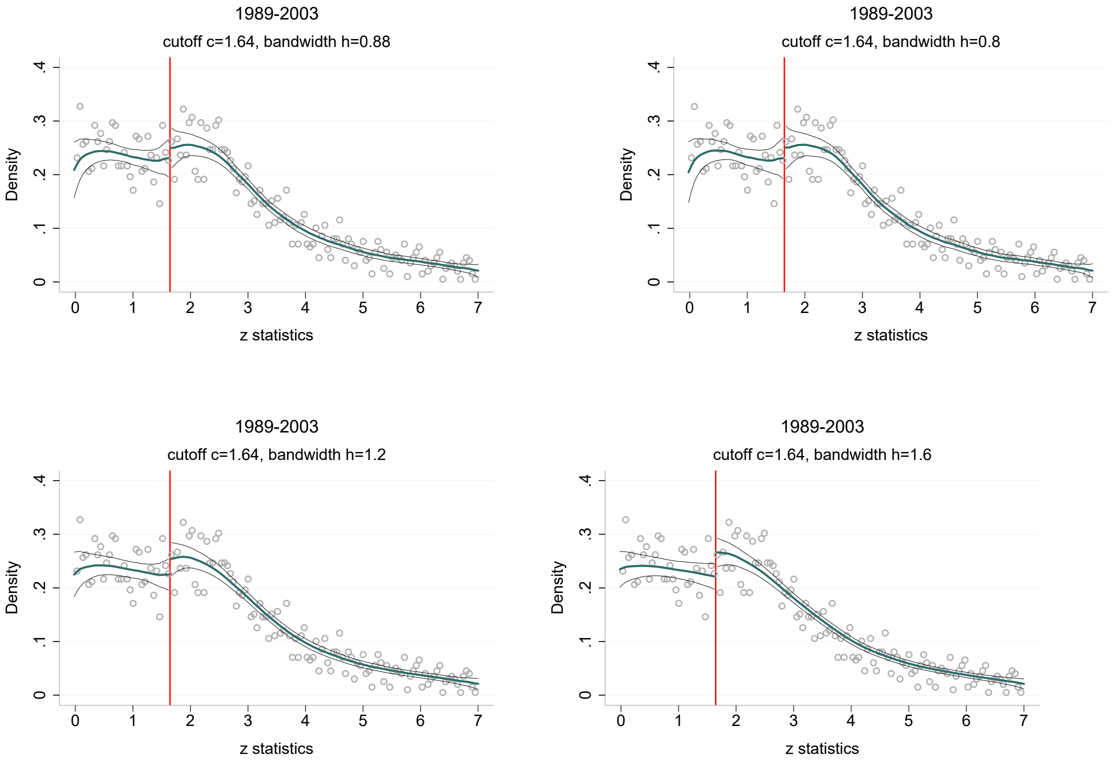

Fig S12. Discontinuity plots, $c = 1.64$, varying bandwidth, 1989-2003
The graphs show the results of the discontinuity estimation by applying the McCrary algorithm for the 10 significance level for unweighted data. We vary the bandwidth h of the local linear regression. The upper left graph shows the results for the default bandwidth, the other graphs show the results for $h = \{0.8, 1.2, 1.6\}$. The graphs plot the distribution (grey circles) and the local linear density estimation (emerald line) with the respective 95% confidence band.

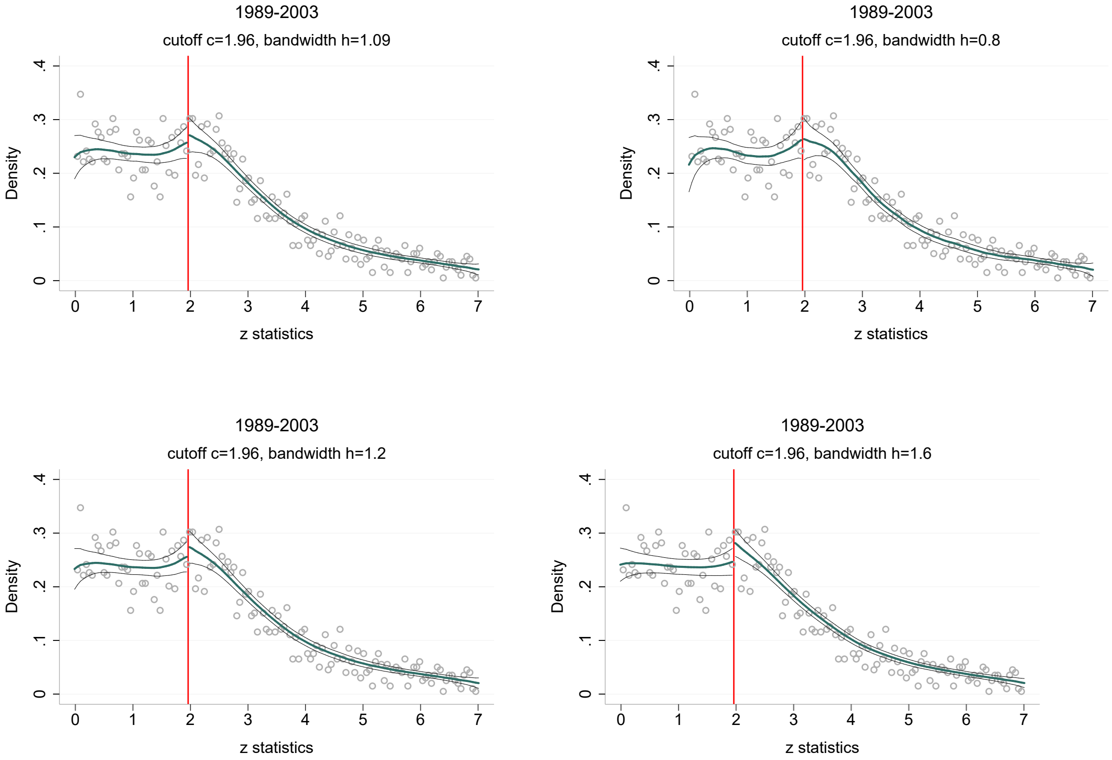

Fig S13. Discontinuity plots, $c = 1.96$, varying bandwidth, 1989-2003
The graphs show the results of the discontinuity estimation by applying the McCrary algorithm for the 5 significance level for unweighted data. We vary the bandwidth h of the local linear regression. The upper left graph shows the results for the default bandwidth, the other graphs show the results for $h = \{0.8, 1.2, 1.6\}$. The graphs plot the distribution (grey circles) and the local linear density estimation (emerald line) with the respective 95% confidence band.

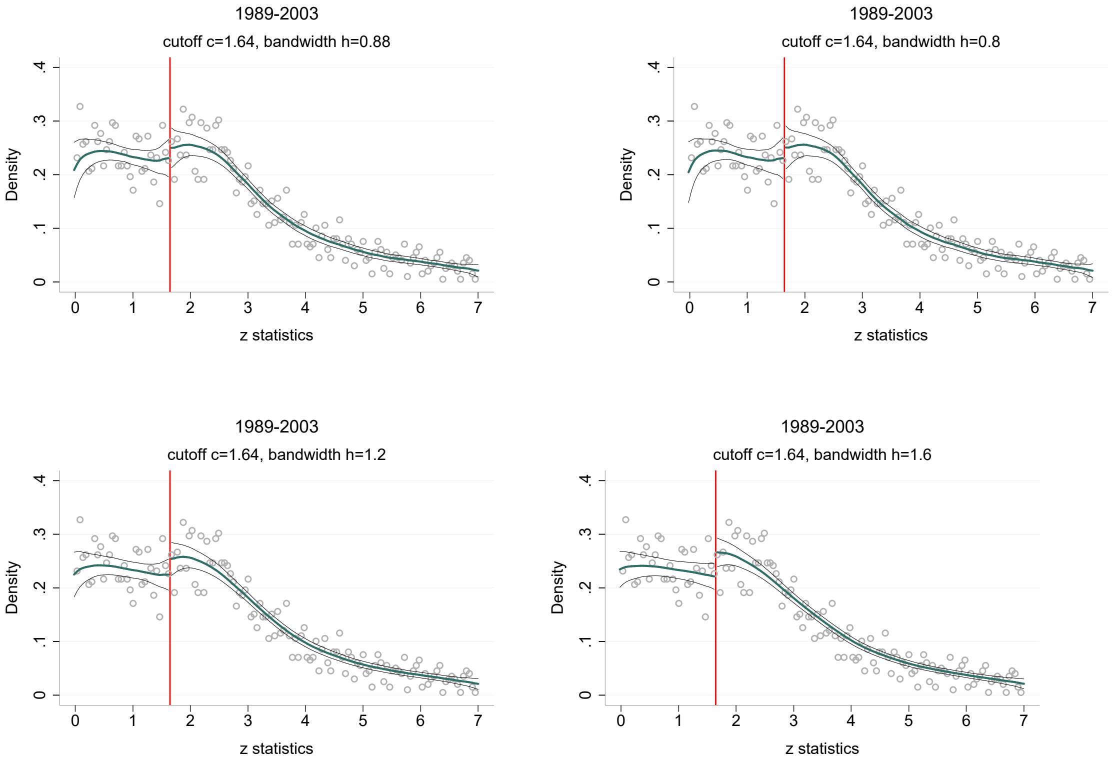

Fig S14. Discontinuity plots, $c = 2.58$, varying bandwidth, 1989-2003
The graphs show the results of the discontinuity estimation by applying the McCrary algorithm for the 1 significance level for unweighted data. We vary the bandwidth h of the local linear regression. The upper left graph shows the results for the default bandwidth, the other graphs show the results for $h = \{0.8, 1.2, 1.6\}$. The graphs plot the distribution (grey circles) and the local linear density estimation (emerald line) with the respective 95% confidence band.

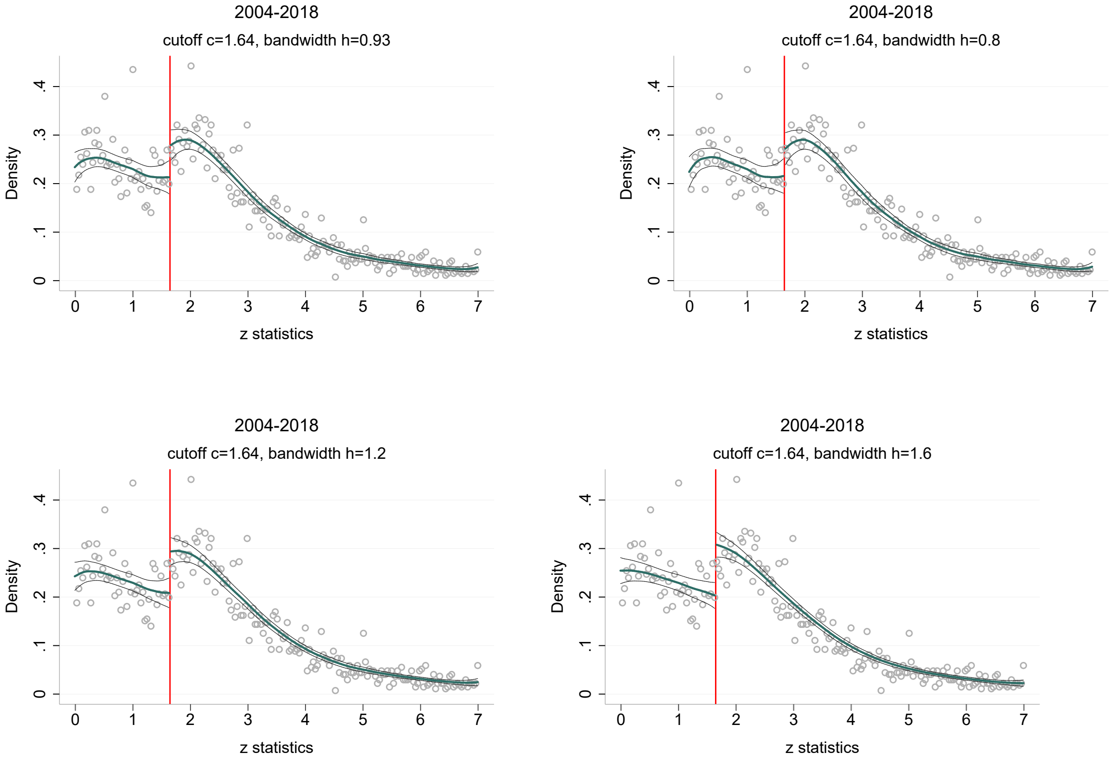

Fig S15. Discontinuity plots, $c = 1.64$, varying bandwidth, 2004-2018
The graphs show the results of the discontinuity estimation by applying the McCrary algorithm for the 10 significance level for unweighted data. We vary the bandwidth h of the local linear regression. The upper left graph shows the results for the default bandwidth, the other graphs show the results for $h = \{0.8, 1.2, 1.6\}$. The graphs plot the distribution (grey circles) and the local linear density estimation (emerald line) with the respective 95% confidence band.

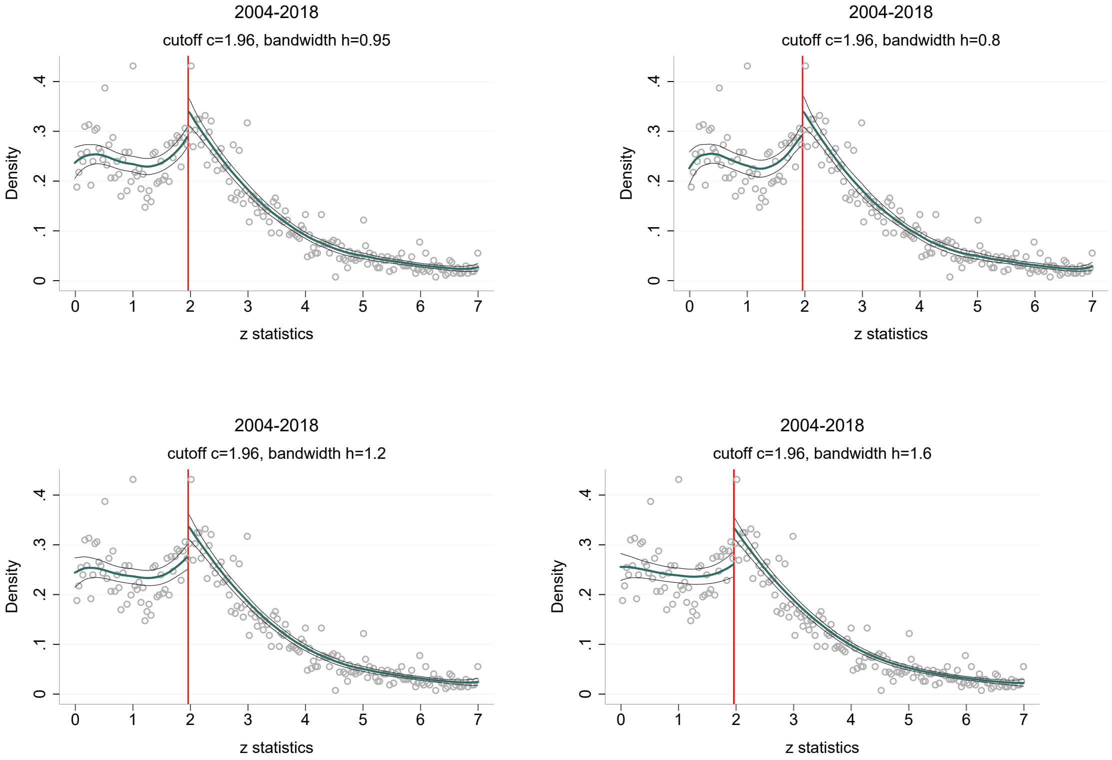

Fig S16. Discontinuity plots, $c = 1.96$, varying bandwidth, 2004-2018
The graphs show the results of the discontinuity estimation by applying the McCrary algorithm for the 5 significance level for unweighted data. We vary the bandwidth h of the local linear regression. The upper left graph shows the results for the default bandwidth, the other graphs show the results for $h = \{0.8, 1.2, 1.6\}$. The graphs plot the distribution (grey circles) and the local linear density estimation (emerald line) with the respective 95% confidence band.

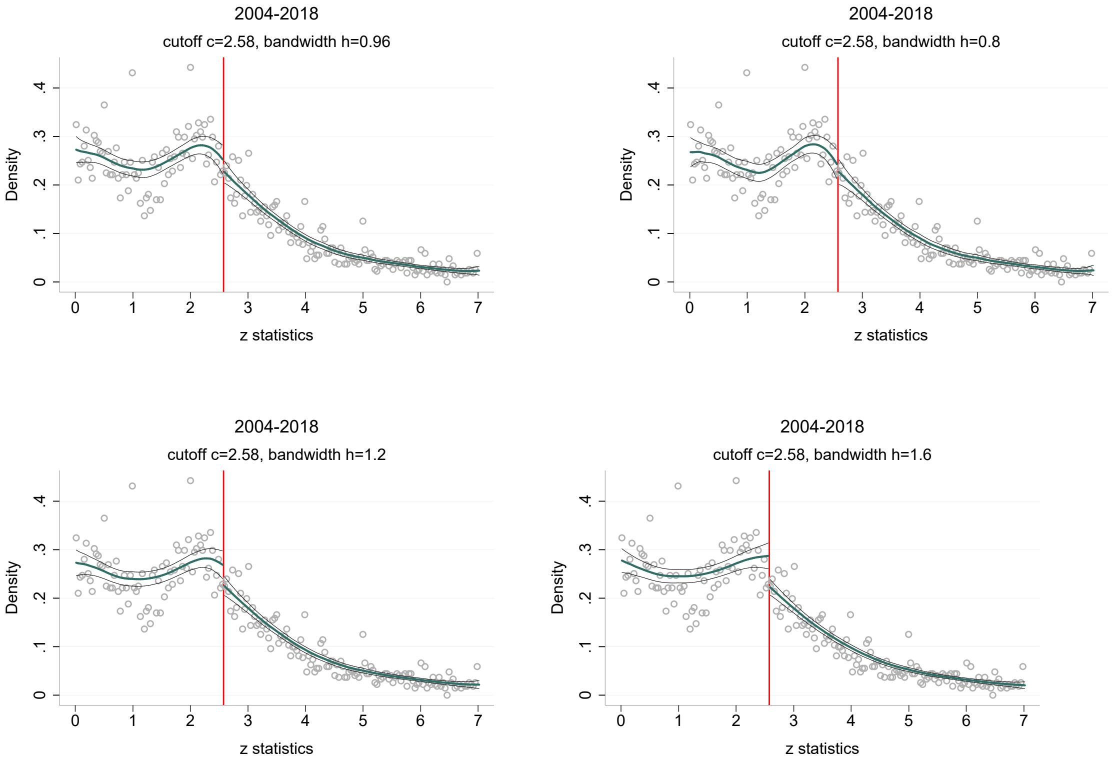

Fig S17. Discontinuity plots, $c = 2.58$, varying bandwidth, 2004-2018
The graphs show the results of the discontinuity estimation by applying the McCrary algorithm for the 1 significance level for unweighted data. We vary the bandwidth h of the local linear regression. The upper left graph shows the results for the default bandwidth, the other graphs show the results for $h = \{0.8, 1.2, 1.6\}$. The graphs plot the distribution (grey circles) and the local linear density estimation (emerald line) with the respective 95% confidence band.

3 Supplementary analyses

3.1 Competing for publications in the *QJE*

Complementary to our analyses on publication bias, we report further trends in publication metrics of the *QJE*. We will focus on two objectives: 1) reconstruction of the development of the acceptance rate for submissions to *QJE*, and 2) documentation of the change in publication volume of the *QJE*. Both will help us to qualitatively evaluate how the competition for publications in *QJE* has changed over time.

We report the acceptance rate and the number of submissions for *QJE* using data provided by [1]. Missing information has been interpolated. Figure S20 shows that the number of submissions increased significantly over time whereas the acceptance rate decreased notably. In the early 1970s about 400 papers were submitted per year. With an acceptance rate just over ten percent about 40 articles each year were accepted. Moving to the mid-1990s, about 6.5% of 600 submissions were published, which again yields about 40 publications. Surprisingly, this number does not change for the recent years. For the last observation, 2011, just over three percent of about 1300 manuscripts were published. This shows that the acceptance rate did not decrease to such low levels because the journal *intentionally* became more selective, but rather because the journal received substantially more submissions over time. This illustrates increasing pressures to publish in high quantity (number of submissions), with high quality (submissions at top journals) and indicates increasing competition (declining acceptance rates to very small levels).

The above mentioned indications for increasing publication pressures are supported by our analyses of the publication volume over time. Figure S21 plots the number of articles and pages published per year as well as the length of published articles over time. Over the last six decades the number of publications per year remained quite stable with about 40 articles per year. Still, the number of total pages per year increased remarkably from about 700 yearly pages between 1960 and 1980 to around 2000 pages in recent years. This can be attributed to the increased average length of the articles. Whereas an article had about ten to fifteen pages in the 1970s and 1980s, the average length increased steadily to 48 pages, which is more than three times as many. This pays tribute to the increasing complexity of the empirical analyses, and once more illustrates that the requirements for the necessary amount of material to warrant a sufficient contribution have substantially increased over time. In other words, it becomes increasingly harder for scientists to meet the expectations for publishing in top journals.

We, further discuss three detailed peculiarities in the trend data on acceptance rates and publication volume. *First*, in 1980 twice as many articles as usual were published. This coincides with the first-time installation of an editorial board and the fact that the *QJE* published two volumes in this particular year.[2] *Second*, in 1985 the editors of the *QJE* published a *Manifesto* announcing that they will "publish 50 percent more pages than usual this year" aiming to reduce "backlog" [2, p. iii]. Figure S21 suggests that they kept their word. *Third*, significantly more articles were published in 1991 as well. Most likely, this can be attributed to a large-scale project of the National Bureau of Economic Research and the Harvard University that ended in 1991 with reports being published in the *QJE* [3]. Taking these observations into account, we reconsider the acceptance rate from Figure S20 that we a priori linearly interpolated between 1977 and 1989 due to missing data. Using the interpolated submissions and the *actual* number of published articles we recalcu-

[2]Additionally to articles published in the *QJE* we also collected the front matters –they usually report the current editor(s)– of all volumes from 1959 to 2018 to get an overview on editorial changes.

lated the acceptance rate.[3] This yields an acceptance rate of about 20% in 1980, implying that almost every fourth submitted manuscript was eventually published. In 1985, the acceptance rate still amounts to 14% which is higher than in adjacent years. For the year 1991, no notable change in the acceptance rate results.

These peculiarities aside, the trend towards lower acceptance rates at *QJE* is in line with changes in other high ranked economics journals. [1] show that most of these journals faced a significant increase in submissions resulting simultaneously in substantially reduced acceptance rates.[4] Aggregating the results of all top journals, the publications to submissions ratio is about 0.10 (300 : 2900) in 1990 and declines to 0.06 ($320 : 5400 = 0.06$) in 2012. Sole exception is the *AER* that reacted to increasing submissions by publishing more articles per year, thereby maintaining an above average acceptance rate of 7.9% in recent years. Overall, these results highlight the increased competition for publications in highly ranked economics journals among scientists. Publications in these journals are very attractive since they usually reach a wider readership and, thus, higher citation counts. It is, therefore, plausible to assume that publication bias is, first, higher in top journals than in medium ranked journals, and, second, increased over time.

[3]To calculate the acceptance rate we follow the procedure of [1] and divide the number of published articles by the average of submissions in the two previous years.

[4]The authors analyzed the journals *QJE*, *AER*, *JPE*, *ECA* and *Review of Economic Studies.*

3.2 Analyses with the caliper test

The caliper test

Since we use a regression discontinuity approach, which has rarely been used before, we want to make sure that our results also hold when using another, more conventional method. Even though there is not yet a "standard" procedure to investigate publication bias in the case of heterogeneous effects, several authors used the so-called *Caliper* test (e.g., [4, 5, 6]) in previous studies.

Just as the McCrary procedure, the caliper test is based on the assumption that, though the exact distribution of test scores is unknown, it should be continuous and without any jumpsn at the thresholds of significance [6]. But, whereas the McCrary procedure uses the entire distribution, thereby giving more weight to the areas that are closer to the respective level of significance, the caliper procedure only focuses on a small local area around the thresholds of statistical significance. It compares the occurrence of test statistics just above and just below the critical threshold of significance (i.e. 1.96 for the two-sided significance level of $\alpha = 0.05$), and is based on the assumption that in absence of publication bias the frequency of test statistics in narrow, equal-sized intervals just above and just below the critical threshold should not differ. Thus, in contrast, a substantial over-representation of observations in the interval just above the critical threshold is regarded as evidence for publication bias.

Therefore, under the null hypothesis of no publication bias, the intervals

$$(c - \epsilon, c] \text{ and } (c, c + \epsilon],$$

where c is the respective level of significance (such as 1.65 for $\alpha = 0.10$, 1.96 for $\alpha = 0.05$, and 2.58 for $\alpha = 0.01$) and ϵ the interval width, should be equally likely. More technical, if we observe n test statistics in the entire range $c \pm \epsilon$ the number of test statistics that fall in the upper interval $(c; c+\epsilon]$ follows a binomial distribution with $(n, p = 0.5)$. The parameter ϵ is a percentage value and denotes the size of the caliper interval. The smaller we choose ϵ, the closer we approach the critical value, thus providing a very strict and precise test of publication bias. However, small caliper intervals yield only small interval sample sizes and therefore have low power. Testing for the different caliper widths, e.g. 5%, 10% and 15%, and comparing the results offers a trade-off between sample size and statistical power.

Despite its major strength of requiring only few distributional assumptions, there are certain drawbacks associated with the caliper test. The caliper test usually involves multiple simultaneous analyses since the caliper sizes are varied. Smaller caliper intervals are always included in larger caliper intervals and researchers would then face a multiple testing problem [4]. However, there are correction methods that may account for that [7]. Further, the test involves a substantial reduction in sample size because only those test statistics very close to the threshold of significance are used for the test. This may eventually result in low power. Additionally, for large interval sizes, hence large ϵ, the caliper intervals from different significance levels will overlap. So it may happen, that a coefficient is in the upper caliper interval of the 10 percent significance level as well as in the lower caliper interval of the 5 percent significance level. In case of doubt, narrower intervals should therefore be preferred.

Table S3 provides an overview of the caliper intervals for which we will compare the prevalence of test statistics for the different significance levels. We chose caliper sizes of $\epsilon = 5\%$, $\epsilon = 10\%$ and $\epsilon = 15\%$.

In what follows, we will, first, report cross-sectional caliper results to get an overview of the overall distribution of test statistics just below and just above the respective significance levels.

Table S3. Overview: Caliper Intervals

	5% caliper		10% caliper		15% caliper	
	UC	OC	UC	OC	UC	OC
10% level z=1.645	(1.563,1.645]	(1.645,1.727]	(1.48,1.645]	(1.645,1.809][a]	(1.398,1.645]	(1.645,1.892][b]
5% level z=1.960	(1.862,1.960]	(1.960,2.058]	(1.764,1.960][a]	(1.960,2.156]	(1.666,1.960][b]	(1.960,2.254][c]
1% level z=2.576	(2.447,2.576]	(2.576,2.705]	(2.318,2.576]	(2.576,2.833]	(2.189,2.576][c]	(2.576,2.962]

Note: UC - under caliper, OC - over caliper. The table shows the interval boundaries at the 10, 5, and 1 percent significance level for varying caliper sizes. [a],[b],[c] - intervals sharing the same superscript do overlap.

Second, we will construct *moving caliper ratios* just as we constructed *moving discontinuities* to investigate time trends.

Cross-section

We start with a cross-sectional caliper analysis. Table S4 reports the number of test statistics that fall in the upper (over caliper) and lower (under caliper) interval for the 10, 5, and 1 percent significance level for varying caliper widths.

For the 10 percent significance level there is a clear over-representation of test statistics in the over caliper. The distribution significantly differs from a uniform distribution for all caliper sizes with at least $p < 0.10$. For the narrowest interval, the 5% caliper, we observe 13.3% more test statistics in the over caliper than in the under caliper. For the largest interval, the 15% caliper, there is an over-representation of even 21.2%. We find similar patterns for the 5 percent significance level. For all caliper sizes, there are significantly more test statistics in the over than in the under caliper (at least $p < 0.07$). The over-representation ranges from 15.6% for the smallest caliper to 8.4% for the largest caliper. In contrast, we find no over-representation for the 1 percent significance level, where there even are more test statistics in the under than in the over caliper.

Table S4. Cross-sectional caliper counts

	**10%-significance level**			
	OC	UC	OC/UC	*p val*
5%-caliper	264	233	1.133	0.089
10%-caliper	547	467	1.171	0.007
15%-caliper	812	670	1.212	0.000
	**5%-significance level**			
	OC	UC	OC/UC	*p val*
5%-caliper	401	347	1.156	0.026
10%-caliper	741	683	1.085	0.065
15%-caliper	1082	998	1.084	0.034
	**1%-significance level**			
	OC	UC	OC/UC	*p val*
5%-caliper	358	406	0.882	0.962
10%-caliper	679	836	0.812	1.000
15%-caliper	974	1255	0.776	1.000

Note: OC - over caliper, UC - under caliper. The *p val* is based on a one-sided binomial test with $(n, p = 0.5)$, whereas n is the number of test statistics in the over and under caliper $OC + UC$. Hence, we test whether the proportion of the over caliper significantly exceeds 50%.

Overall, these results confirm the results from our cross-sectional discontinuity analysis, where we find similar patterns.

Time trend

We further examine whether our longitudinal results for the *moving discontinuities* also hold when we transfer the principle to the caliper approach. We, therefore, equally construct *moving caliper ratios* for our preferred specification with 15 year windows:

$$
\begin{aligned}
ratio_{1959} &= OC_{Z_{1959},Z_{1960},\ldots,Z_{1973}}/UC_{Z_{1959},Z_{1960},\ldots,Z_{1973}}\\
ratio_{1960} &= OC_{Z_{1960},Z_{1961},\ldots,Z_{1974}}/UC_{Z_{1960},Z_{1961},\ldots,Z_{1974}}\\
&\vdots\\
ratio_{2004} &= OC_{Z_{2004},Z_{2005},\ldots,Z_{2018}}/UC_{Z_{2004},Z_{2005},\ldots,Z_{2018}}
\end{aligned}
$$

where Z_y is the set of test statistics extracted in year y and OC_{Z_y} (UC_{Z_y}) then refers to the number of test statistics in the over (under) caliper. The value $ratio_j$ then represents the ratio of the number of test statistics in the over caliper versus the number of test statistics in the under caliper for the 15-year interval starting with year j.

We report the results in Figure S22. Panel A, B and C refer to 10, 5, and 1 percent significance level, respectively. Again, we find patterns very similar to our results from the *moving discontinuities* (article Figure 5). For the 10 percent significance level we observe a pattern that strikingly resembles the McCrary results. The over-representation of test statistics in the over caliper is large in the first half of the observation period –depending on the caliper size, the over-representation ranges between 50% and 300%– and drops notably around 1990. Later, it slowly increases again to a significant over-representation of around 20%. For the 5 percent significance level there is no significant over-representation of the over caliper in earlier years. However, for recent years we find a significant over-representation of around 15%. In contrast, we do not find a significant over-representation for any interval at the 1 percent significance level.

Subfig A: 10 percent significance level ($c = 1.64$)

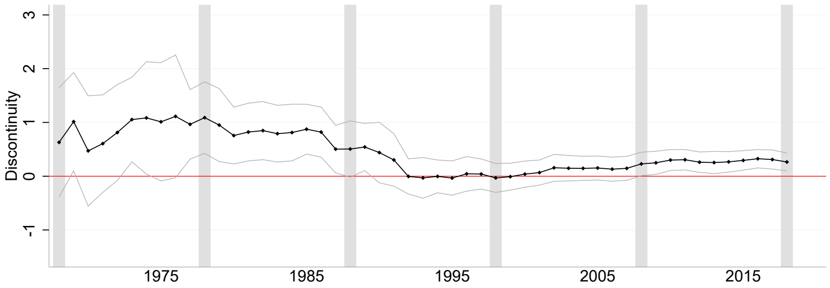

Subfig B: 5 percent significance level ($c = 1.96$)

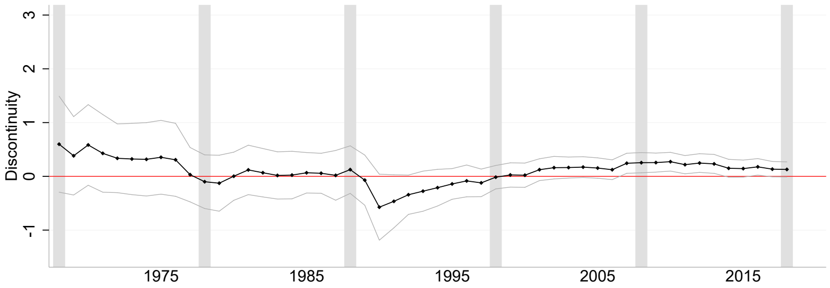

Subfig C: 1 percent significance level ($c = 2.58$)

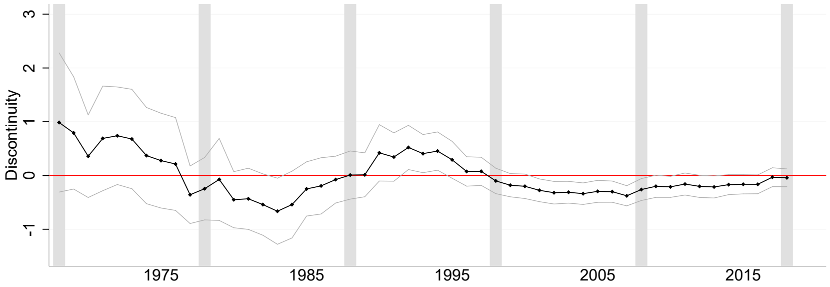

Fig S18. Moving discontinuity estimates for $k = 10$ *years* time windows

The graphs plot *moving discontinuity* estimates for the 10, 5 and 1 percent significance level for $k = 10$ *years* time windows. Each dot is the result of a single discontinuity estimation. The x axis marks the end point of the respective intervals. For example, the first dot reports the estimate for the interval 1959–1968, the second dot reports the estimate for the interval 1960–1969 and so forth. Adjacent dots relate to intervals that overlap by 9 years each. The outer lines denote the 95% confidence interval and the gray bands highlight the estimation results for disjoint intervals.

Subfig A: 10 percent significance level ($c = 1.64$)

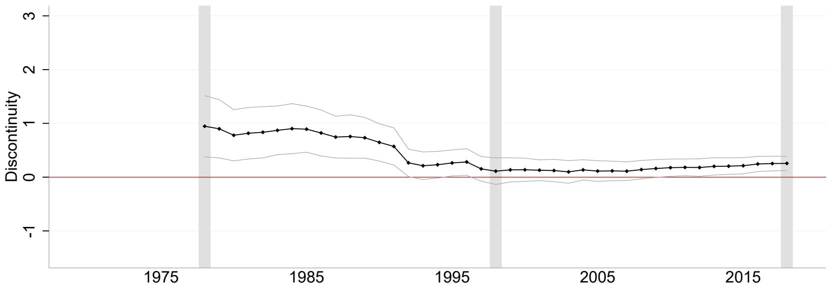

Subfig B: 5 percent significance level ($c = 1.96$)

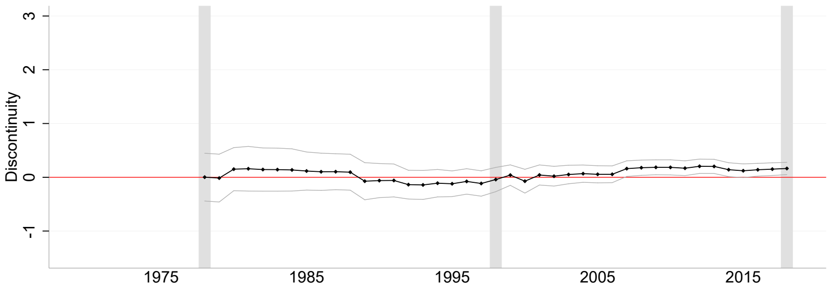

Subfig C: 1 percent significance level ($c = 2.58$)

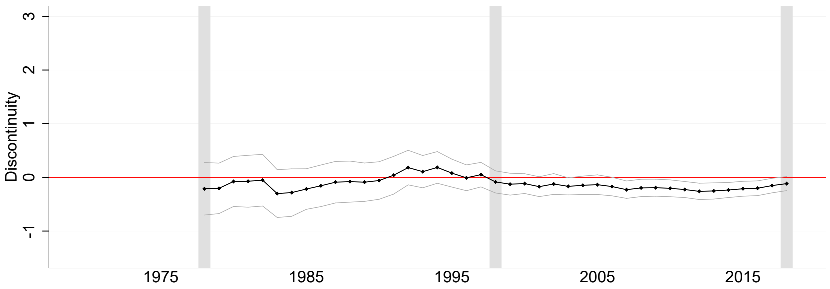

Fig S19. Moving discontinuity estimates for $k = 20$ *years* time windows

The graphs plot *moving discontinuity* estimates for the 10, 5 and 1 percent significance level for $k = 20$ *years* time windows. Each dot is the result of a single discontinuity estimation. The x axis marks the end point of the respective intervals. For example, the first dot reports the estimate for the interval 1959–1978, the second dot reports the estimate for the interval 1960–1979 and so forth. The outer lines denote the 95% confidence interval and the gray bands highlight the estimation results for disjoint intervals.

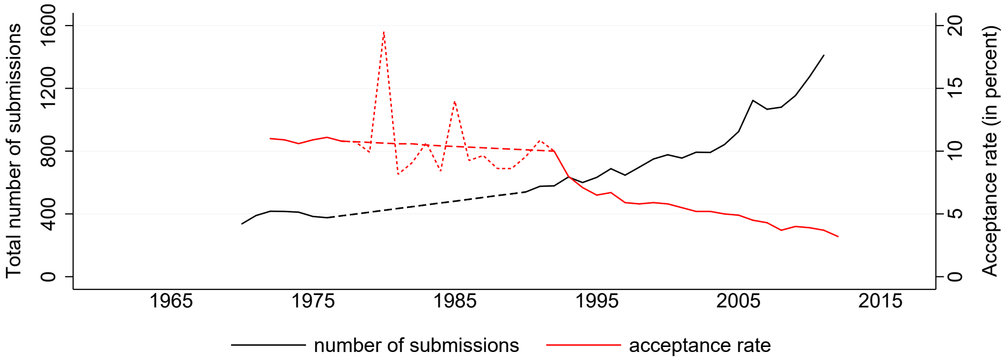

Fig S20. Submissions and acceptance rate over time, 1959 – 2018
Data Source: [1] and own calculations. The left-hand axis refers to the number of submissions per year to the *QJE* (black line) and the right-hand axis refers to the corresponding acceptance rate (red line). The acceptance rate is based on articles published in the respective year in comparison to lagged submissions from the two previous years. For the years before before 1970, the years after 2012 and the years 1977 to 1989 no submission data is available. Between 1977 and 1989 we linearly interpolated the missing values for the number of submissions and the acceptance rate (dashed segments). For the acceptance rate we also recalculated the values using the linearly interpolated submission and the actual number of publications (dotted segment), see text for detailed descriptions.

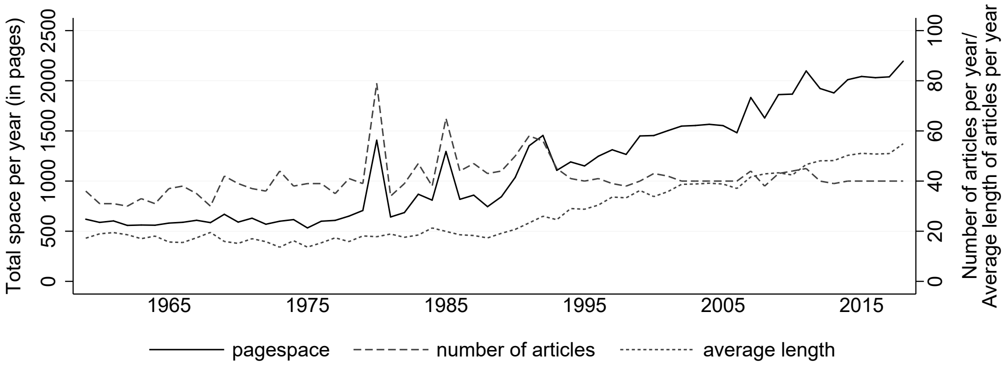

Fig S21. Publication volume over time, 1959 – 2018
The graph plots the overall publication volume (measured by total number of pages per year) against the number of articles published per year and their average length. We excluded discussion items (comments, notes, replies, etc.) for this analysis. The left-hand axis refers to the total number of pages (solid line) published per year. The right-hand axis refers to the number of articles published per year (dashed line) as well as the average length of the articles (dotted line).

Subfig A: 10 percent significance level ($c = 1.64$)

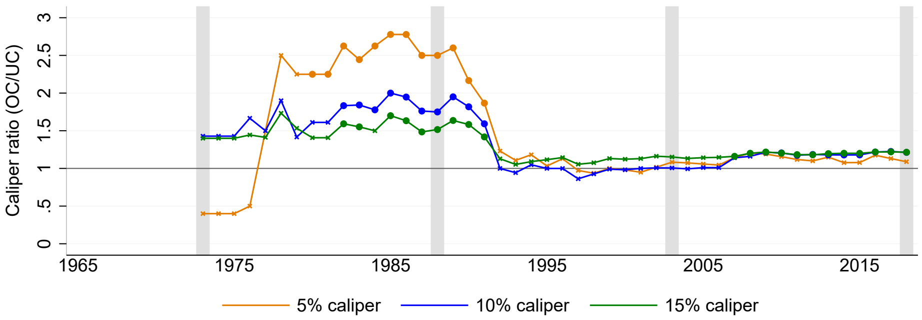

Subfig B: 5 percent significance level ($c = 1.96$)

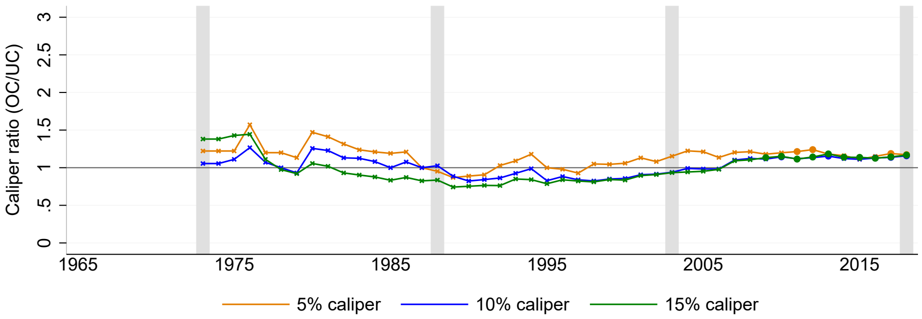

Subfig C: 1 percent significance level ($c = 2.58$)

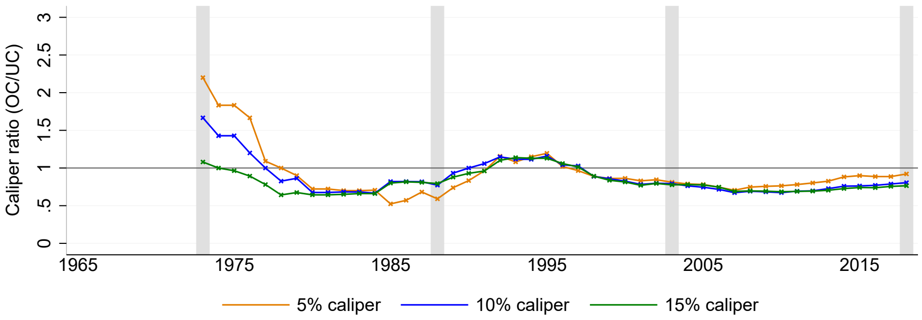

Fig S22. Moving caliper ratios for $k = 15$ *years* time windows
The graphs plot *moving caliper ratios* for the 10 percent, 5 percent and 1 percent significance level for $k = 15$ *years* time window. Values above 1 indicate an over-representation of results in the over caliper. The x- axis marks the end point of the respective intervals. For example, the first dot reports the caliper ratio for the interval 1959–1968, the second dot reports the caliper ratio for the interval 1960–1969 and so forth. The gray bands highlight the caliper ratios for disjoint intervals. Dots (•) indicate intervals where the the number of test statistics in the over caliper significantly exceeds 50% at the 95% confidence level. A cross (×) indicates intervals, where there is no significant over-representation.

References

[1] Card D, DellaVigna S. Nine Facts about Top Journals in Economics. Journal of Economic Literature. 2013;51(1):144–161.

[2] Blanchard OJ, Maskin ES, Summers LH. Manifesto. The Quarterly Journal of Economics. 1985;100(1):iii. doi:10.1093/qje/100.1.iii.

[3] Barro RJ, Romer PM. Introduction. The Quarterly Journal of Economics. 1991;106(2):i. doi:10.1093/qje/106.2.i.

[4] Auspurg K, Hinz T, Schneck A. Ausmaß und Risikofaktoren des Publication Bias in der deutschen Soziologie. Kölner Zeitschrift für Soziologie und Sozialpsychologie. 2014;66:549–573.

[5] Berning CC, Weiß B. Publication bias in the German social sciences: an application of the caliper test to three top-tier German social science journals. Quality & Quantity. 2016;50:901–917.

[6] Gerber A, Malhotra N. Do Statistical Reporting Standards Affect What is Published? Publication Bias in Two Leading Political Science Journals. Quarterly Journal of Political Science. 2008;3:313–326.

[7] Holm S. A Simple Sequentially Rejective Multiple Test Procedure. Scandinavian Journal of Statistics. 1979;6(2):65–70.
